# Ultrafast Kerr Spectroscopy Reveals Bulk‐Like Solvent Dynamics in Concentrated LiTFSI–Acetonitrile Electrolytes

**DOI:** 10.1002/cplu.202500579

**Published:** 2026-01-15

**Authors:** Yousaf Shah, Bruno A. Cândido, Pedro Migowski, Stephen R. Meech, Ismael A. Heisler

**Affiliations:** ^1^ Instituto de Física Universidade Federal do Rio Grande do Sul ‐ UFRGS Porto Brazil; ^2^ Instituto de Química Universidade Federal do Rio Grande do Sul ‐ UFRGS Porto Brazil; ^3^ School of Chemistry Norwich Research Park University of East Anglia Norwich UK

**Keywords:** acetonitrile, dynamics, electrolytes, LiTFSI, spectroscopy

## Abstract

Electrolyte solutions are vital to energy storage devices, significantly influencing their capacity, safety, and cost efficiency. Lithium salts based on multidentate anions have shown remarkable potential in energy storage, particularly when dissolved in acetonitrile. These solutions exhibit exceptionally high ionic conductivities, even for concentrations above the standard 1 mol L^−1^ solutions. To directly probe bulk solvent and solvation shell dynamics in lithium salt solutions, the ultrafast optical Kerr effect (OKE) method is utilized. We investigate the microscopic dynamics of LiTFSI (lithium bis (trifluoromethanesulfonyl) imide) solutions at various concentrations in acetonitrile. The measured data, combined with a global analysis method, reveal that the solvent remains highly dynamic and nearly bulk‐like, even at high concentrations where a significantly reduced number of solvent molecules are available to solvate the cations in solution. These findings support recent explanations as to why acetonitrile‐based electrolyte solutions exhibit higher conductivity compared to, for instance, other nonaqueous electrolyte solutions. In electrolytes based on acetonitrile, a greater proportion of free solvent molecules results in lower overall viscosity. An abundance of uncoordinated solvent molecules facilitates higher ion conduction, compared with the more limited ion mobility observed in other LiTFSI electrolyte systems.

## Graphical abstract

Ultrafast OHD‐OKE spectroscopy is used to directly probe the intermolecular and solvation‐shell dynamics of LiTFSI–acetonitrile electrolytes. Despite strong ion–solvent interactions at high salt concentrations, the measurements reveal a persistent population of highly dynamic, bulk‐like ACN molecules, together with distinct slower components arising from coordinated solvent and intrinsic anion polarizability relaxation. These results provide microscopic, time‐domain evidence explaining why ACN‐based electrolytes maintain low viscosity and high ionic conductivity even at elevated LiTFSI loadings.

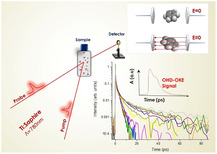

## 1 Introduction

Concentrated solutions of lithium (Li) salts represent a particularly promising class of next‐generation liquid electrolytes [1, 2]. Nonaqueous electrolyte solutions are widely used in electrochemical energy storage due to their distinctive physicochemical characteristics, which include a broad electrochemical stability window, strong thermal stability, and extended liquid phase range [3, 4, 5, 6]. Lithium‐ion batteries (LIBs), commercialized first in 1991, are now standard in portable electronics and are growing in popularity in the market for electric vehicles [7, 8, 9]. Since LIBs are nearing their energy density limits, further advancements are needed to enhance capacity, safety, performance, electrochemical stability, lifespan, and cost efficiency [10, 11]. The electrolyte plays a crucial role in these developments, impacting both capacity and safety. While various electrolytes have been explored to improve these aspects, challenges related to electrochemical stability persist [7, 12, 13, 14, 15, 16].

To address some of these problems, a new generation of highly concentrated electrolytes (HCE) is increasingly being studied [2, 15, 17, 18, 19]. As with previously known electrolytes, HCEs consist of Li salts dissolved in high dielectric solvents, but with concentrations well above one molar. Although the cation is clearly the key component (from the perspective of energy storage capacity), studies have shown that the molecular characteristics of the anion and solvent are also critical, significantly affecting electrolyte properties such as conductivity and electrochemical stability [14]. From these studies, it emerged that multidentate anions with large charge delocalization produce improved electrolyte performance [9]. A class of anions that possess such properties are bis(trifluoromethanesulfonyl)imide (TFSI^−^) and its variants. With regard to the solvents, those containing polar groups such as carbonyl (C=O), nitrile (C≡N), sulfonyl (S=O), and ether linkages (–O–) have been considered as candidates for nonaqueous electrolytes. Among them, acetonitrile (ACN) stands out as one of the most oxidation‐tolerant organic solvents, which may enable the use of high‐voltage electrodes (>5 V) [20, 21].

To better understand the structure and dynamics in electrolyte solutions and explain the significant transport numbers in HCEs and its correlation with structure and dynamics of the molecules and ions in the electrolyte solutions, a variety of techniques have been utilized. These include neutron, X‐ray and dynamic light scattering, nuclear magnetic resonance (NMR), and infrared and Raman spectroscopy [22, 23, 24, 25, 26, 27, 28, 29]. It is known that lithium ions interact with both solvent and anions, creating extended ionic networks that are highly fluid and dynamic, on timescales which can range from femto to picoseconds [25]. IR methods, such as 2D‐IR, probe dynamics in such electrolyte solutions by exciting a vibrational mode in the solvent such as the nitrile stretching mode of ACN, which is a sensitive marker for cation‐solvent and ion–ion interactions [26, 30]. Recent 2D‐IR studies on LiTFSI–ACN systems revealed ultrafast exchange processes within solvation structures [31]. A limitation of these methods is that the translational and/or orientational motions of the molecules and ionic structures must be faster than the vibrational mode excited state lifetime for it to be successfully resolved. An alternative approach, complementary to IR methods, is to probe the solvent molecules directly in the ground state, through a Raman process. In this regard, here we apply the ultrafast optically heterodyne detected optical Kerr effect (OHD‐OKE) and show that it has the ability to provide unique and detailed information, offering insights into the dynamics and interactions within the electrolyte solutions [32]. Related ultrafast spectroscopy studies on concentrated ionic liquids and LiTFSI‐based electrolytes have likewise revealed slow anion polarization modes and heterogeneous solvent relaxation. Our present results extend such observations to acetonitrile electrolytes under well‐defined concentration control [27, 33, 34].

In essence, OHD‐OKE measures the relaxation of a transient polarizability anisotropy induced by a linearly polarized ultrafast pump pulse in a fluid [35]. A significant advantage of the method is that it provides direct time‐resolved dynamical information of the solvent molecules in bulk, the solvation shell, and ion–solvent interactions, with a high signal‐to‐noise ratio. Furthermore, through a simple Fourier transform procedure of the acquired time domain data, the low‐frequency Raman spectral density can be directly determined, unaffected by the laser's finite pulse width or the thermal population of low‐frequency modes [35]. The Raman spectral density comprises data on low‐frequency Raman active intramolecular modes, molecular orientational relaxation (diffusive and nondiffusive), and intermolecular (interaction‐induced) interactions [36]. All these dynamical mechanisms play a vital role in understanding the molecular dynamics of liquids and can be simulated by molecular dynamics calculations. It is hoped that such detailed microscopic information on the dynamics, arrangements, and interactions among ions and solvent molecules will inform the development of improved electrolytes for LIBs [2, 11]. While IR pump–probe methods have revealed ultrafast solvation dynamics, their observation window is limited by the vibrational lifetimes of the probe modes (CN, CO). In contrast, OHD‐OKE accesses solvent and ion relaxation from sub‐picoseconds up to tens of picoseconds, enabling assignment of both ultrafast and slower dynamical components.

Here, LiTFSI‐ACN electrolytes at various concentrations were studied through OHD‐OKE spectroscopy. It was found that the solvent remains highly dynamic even at quite high salt concentrations. Our findings are consistent with recent explanations that acetonitrile‐based electrolyte solutions exhibit higher conductivity than many other nonaqueous systems, and they provide valuable new insights into the molecular origins of this enhanced conductivity [37, 38].

## 2 Results and Discussion

The neat ACN OHD‐OKE response has been investigated experimentally and theoretically before by different groups [36, 39, 40, 41, 42]. The current understanding is that the sub‐picosecond dynamics can be assigned to librational motion, which is an oscillatory or restricted (by neighboring sites) rotational motion of the solvent molecules around a fixed equilibrium position defined by their neighbors, plus an interaction‐induced polarizability relaxation term [35]. The longer, typically picosecond relaxation, is assigned to diffusive reorientational motion of the solvent molecules in solution. As shown in Figure S1 (semilog plot), after an initial fast and multicomponent decay, the polarizability anisotropy relaxation of neat ACN can be fit with an exponential decay with a time constant of 1.65 ps. This result is in good agreement with previous literature, including experimental OHD‐OKE and molecular dynamics results as well as broadband dielectric relaxation studies [42, 43, 44, 45]. In the latter, sub‐picosecond librational and cage‐rattling motions were identified, followed by a collective dielectric relaxation at ~3.3 ps associated with rotational diffusion of ACN dipoles under near‐slip conditions [45]. In this context, the ~0.48 ps relaxation retrieved in our OHD‐OKE analysis corresponds to the interaction‐induced/cage‐rattling contribution, while the 1.65 ps component reflects diffusive reorientation of bulk‐like ACN molecules. The additional slower components observed upon LiTFSI addition that will be discussed next thus represent perturbations of these well‐established solvent dynamics by ion coordination and anion polarizability relaxation.

The model used to fit the data, consisting of a sum of exponential terms, is detailed in the Supporting Information. We tested bi‐, tri‐, and four‐exponential models across all mole fractions. While simpler models reproduce the overall decay, they fail to capture both the sub‐picosecond librational contribution and the slower (>10 ps) tail. Residuals show systematic structure under reduced‐component fits, whereas the four‐exponential model yields the best minimized residuals. In this work, we investigate changes in picosecond‐scale diffusive reorientational dynamics induced by the addition of LiTFSI salt (structure shown in Figure S1) to the solution, with the aim of elucidating the underlying microscopic molecular motions within the solvation shell. The faster, subpicosecond contributions will be mentioned only briefly.

**FIGURE 1:**
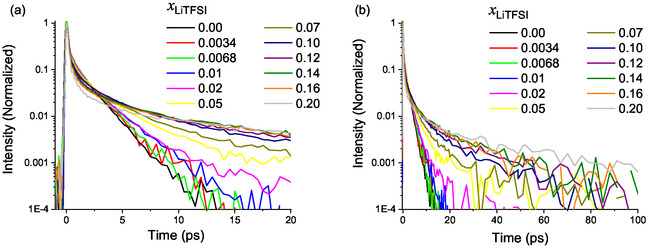
OHD‐OKE curves for various LiTFSI mole fractions for short (a) and long (b) pump‐probe delay times. All amplitudes are normalized to the intensity of the instantaneous electronic (nonresonant) response at time zero.

**FIGURE 2:**
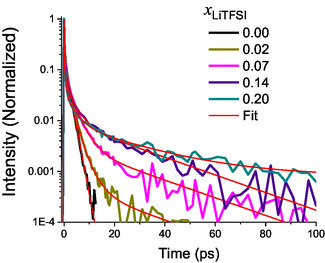
OHD‐OKE curves for a selection of mole fractions and their corresponding fits.

**FIGURE 3:**
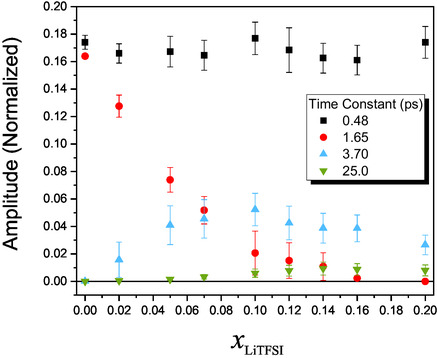
Pre‐exponential factors associated to their corresponding time constants, as a function of solution mole fractions.

Mole fractions were studied up to 0.2, corresponding to approximately four ACN molecules per LiTFSI molecule (i.e., two ACN molecules per ion) [24]. A mole fraction of 0.2 corresponds to a concentration of 2.91 mol/L, which can be considered a highly concentrated electrolyte solution [46]. The measured OHD‐OKE curves are shown in Figure 1 on a semilog graph (for short, Figure 1a, and long time delays, Figure 1b—data were peak normalized at zero time delay). The most prominent effect of increasing the LiTFSI concentration in the ACN‐based electrolyte is the emergence of slower relaxation components, which become progressively more pronounced with increasing salt mole fraction. In order to quantify the dynamical alterations in the different solutions, a fitting procedure equivalent to the one used to fit neat ACN was applied (as described in the SI), except for the inclusion of additional exponential terms to account for the slower relaxation components. All transients were analyzed using a global multiexponential fitting scheme, implemented in Glotaran, in which the characteristic time constants were shared across all mole fractions, while amplitudes were allowed to vary with concentration. This procedure yields a statistically robust set of relaxation times representative of the dominant molecular processes, minimizing overfitting and ensuring self‐consistent interpretation across datasets. Independent fits of each trace yielded *τ*‐values within 10% of those obtained globally, confirming that the global model does not artificially constrain the data [47]. The quality of the fit can be appreciated in Figure 2 for a selection of mole fractions, and in detail for the neat solvent, shown in Figure S1. The whole series could be fit to only four exponential relaxation terms. Figure 3 presents the retrieved exponential amplitudes, or pre‐exponential factors, associated to their corresponding exponential decay time constants. Complete numerical results of the global analysis, including amplitudes and standard deviations, are provided in Table S2. The fastest exponential relaxation decays with a time constant of 0.48 ps. Its amplitude is relatively insensitive to mole fraction change. The second relaxation component, with a time constant of 1.65 ps, has its maximum amplitude for neat ACN. The amplitude of this component then decreases, reaching zero for mole fractions at and above 0.16. The third component, with a time constant of 3.70 ps, shows a small amplitude for a mole fraction of 0.02, goes through a maximum at a mole fraction of 0.1, and then slightly decreases as the salt mole fraction increases. The last component, with a time constant of 25 ps, has an overall small amplitude and starts to contribute for mole fractions above 0.07 and steadily increases up to the highest measured mole fraction, as shown in Figure 3.

The global fit provides valuable insights into the molecular dynamics of the LiTFSI‐ACN electrolytes, enabling the assignment of physical mechanisms underlying the observed relaxation components. The fastest relaxation, with a time constant of 0.48 ps, is present for all the concentrations, with an amplitude that is relatively independent of mole fraction. It is well established, experimentally and theoretically, that most liquids have an interaction‐induced polarizability relaxation contribution, which decays typically with time constants in the range from 0.2 to 0.6 ps [32]. As this time constant is intermediate between fast librational relaxation and the usually slower diffusive reorientational relaxation, it is known as the intermediate relaxation component. Such a contribution is clearly present in neat ACN and remains steadily present as the concentration of the salt in the solution increases. As the solvent content decreases in the various solutions, the ions themselves may introduce an interaction‐induced polarizability relaxation component. Although the time constant may exhibit slight variations across different mole fractions, it can generally be approximated by a value comparable to that observed for the pure solvent. The next exponential relaxation with a time constant of 1.65 ps can be unambiguously assigned to ACN diffusive relaxation, as it is the longest contribution present in the neat solvent. As the salt mole fraction increases, this relaxation component persists, but its amplitude diminishes. This is expected, as the relative number of solvent molecules in close proximity to identical neighbors decreases at higher solute concentrations. However, a notable observation is the persistence of this relaxation component even at a mole fraction of 0.14, where the number of solvent molecules per ion is on average only three. This finding suggests that ACN can still reorient diffusively, as if it were in the bulk, and this relaxation component disappears only at mole fractions above 0.14. To our knowledge, this is the first direct time‐domain demonstration of persistent bulk‐like ACN relaxation at mole fractions as high as 0.14, where only three solvent molecules per LiTFSI remain. Such evidence complements previous structural interpretations based on ion aggregation by providing quantitative dynamical signatures of free solvent behavior [25, 27, 33].

**FIGURE 4:**
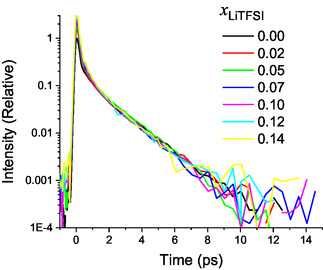
Remaining curves after longest exponential terms are subtracted for the measured data.

To further highlight the presence of a 1.65 ps exponential relaxation component in the data, the longest exponential contributions were subtracted from the experimental curves, and the results for the various mole fractions, up to 0.14, are shown in Figure 4. Due to the different amounts of solvent molecules in the different solutions, the curves were rescaled by a constant arbitrary factor in order to match the neat ACN data. As can be seen in Figure 4, there is a good agreement among the resulting curves, i.e., the resulting subtracted curves overlap well with neat ACN, from 1 ps up to around 10 ps. In the subpicosecond region, extra amplitude due to LiTFSI low‐frequency anisotropic intramolecular vibrational modes appears, and therefore in this region, the curves are mismatched. Specifically, a mode at *ν* ≈ 120 cm^−1^, which is assigned to SNS twisting mode of TFSI^−^ anions has a significant amplitude in this region [48]. The spectral density associated to the OHD‐OKE time domain curves, obtained from the imaginary part of the Fourier transform of the OKE signal divided by the measured autocorrelation at the sample position, is shown in Figure S2 [35]. It clearly shows a peak *ν* ≈ 120 cm^−1^.

For small solute concentrations, it is expected that, on average, the solvent dynamics should not change much from the bulk. However as ions are dissolved in the solution, it is well known that ACN molecules, which have one primary interaction site, given by its nitrile group, tend to coordinate dissolved cations [24]. The TFSI^−^ anions are not coordinated by the solvent, as their solvation primarily relies on hydrogen bonding, which is absent in aprotic solvents like ACN [24, 49]. Instead, structural studies have shown that in concentrated LiTFSI‐ACN solutions essentially all ACN molecules solvate Li^+^, while TFSI^‐^ exists as contact ion pairs and aggregates with Li^+^, forming a polymeric ionic network. Thus, TFSI^−^ remains uncoordinated by ACN, consistent with our assignment of its intrinsic polarizability relaxation [24, 25].

Consequently, a competition arises between the solvent molecules and anions for coordination with the Li^+^ cations in the solution. This coordination slows down ACN diffusive dynamics, as indeed reflected by the appearance of a third slower relaxation component with a time constant of 3.7 ps. This relaxation contribution starts to increase in amplitude, going through a maximum at mole fraction 0.1 and then decreases monotonically (only slightly) with increasing mole fraction. The decrease in amplitude suggests that this component is indeed associated with ACN molecules, as the ACN amount naturally diminishes with an increasing solute mole fraction. However, the observed slowdown in our measurements is relatively modest—only about a factor of two—despite the viscosity increasing by approximately a factor of 30. Here the comparison is between neat solvent, which has a viscosity of 0.35 cP, and mole fraction 0.2, which has a viscosity of 12 cP (the experimentally obtained viscosities are listed in Table S1). Diffusive reorientational dynamics can generally be explained by the Stokes–Einstein–Debye (SED) relation, which predicts a linear relationship between the relaxation time constant and viscosity [50]. However, our results do not follow such SED model, pointing to more complex dynamics within the electrolyte. Future temperature‐dependent OHD‐OKE studies will enable quantitative correlation between relaxation times and macroscopic transport parameters such as viscosity, providing deeper mechanistic insight into deviations from SED behavior.

When lithium salts are dissolved, Li^+^ ions are typically coordinated by solvent molecules in a tetrahedral structure [24]. At a Li^+^:ACN ratio of 1:4, the average number of solvent molecules per cation is indeed close to the coordination number typically required for tetrahedral solvation. However, previous structural studies have shown that full solvation of Li^+^ by ACN occurs only at higher solvent‐to‐salt ratios (≥10:1), whereas at lower ratios TFSI^−^ anions increasingly compete for coordination sites [24, 51]. Thus, even at 1:4, not all Li^+^ centers are fully solvated solely by ACN molecules; instead, a distribution of coordination environments exists in which one or more TFSI^−^ anions replace ACN in the primary solvation shell. This heterogeneous solvation environment is consistent with the dynamical signatures observed in our OHD‐OKE measurements [49]. The persistence of bulk‐like acetonitrile relaxation up to mole fractions of 0.14 suggests a heterogeneous solvation environment, where Li^+^ centers are only partially coordinated by ACN molecules. At high concentrations, competitive coordination by TFSI^−^ anions leads to the formation of contact ion pairs and extended aggregates, effectively releasing a fraction of solvent molecules from the primary solvation shell. These liberated ACN molecules can undergo diffusive reorientation almost as in the bulk phase, explaining the modest slowing of reorientational dynamics compared with the strong viscosity increase. This scenario mirrors the behavior of localized high‐concentration electrolytes (LHCEs), where a noncoordinating diluent decouples ion transport from bulk viscosity. In the present system, the weakly solvating nature of ACN and the high anion polarizability of TFSI^−^ naturally generate a similar effect without the need for an external diluent.

The preferential Li^+^ solvation by its counterion might be one of the mechanisms capable of releasing ACN molecules back to the bulk state, enabling the solvent to remain highly dynamic [37]. Furthermore, as already mentioned, TFSI^−^ anions dissolved in aprotic solvents remain uncoordinated by the solvent molecules, which enables dynamical freedom to the surrounding solvent molecules. For the highest concentrations, other structures may be present such as solvent‐separated ion pairs and aggregates. Despite this, our results demonstrate that ACN molecules remain highly dynamic, even at mole fractions where there are scarcely enough solvent molecules to fully solvate the Li^+^ cations. This supports findings and discussions from recent literature, emphasizing the resilience and dynamic behavior of ACN in such concentrated environments [24, 37, 51].

For the highest mole fractions studied (0.16 and 0.2), there are only 5 and 4 ACN molecules per LiTFSI, respectively. For these solutions, the data could be fit by the 3.7 and 25 ps relaxation components (apart from the subpicosecond interaction‐induced component). As already assigned, the 3.7 ps component is related to slower ACN molecules in the Li^+^ solvation shell. The slowest relaxation (~25 ps) is assigned to the intrinsic polarizability relaxation of the TFSI^−^ anion, consistent with previous studies of ionic liquid solutions. We emphasize that this assignment does not imply specific anion–solvent coordination by ACN (e.g., hydrogen bonding). Instead, the ACN medium provides dielectric stabilization, while the ~25 ps response reflects the internal polarizability dynamics of the anion itself. To provide further support for this assignment, we analyzed the amplitude of the low‐frequency TFSI^−^ SNS twisting mode (120 cm^−1^) which was determined by analyzing the spectral density associated to the time domain OHD‐OKE curves, shown in Figure S2. The amplitude of the slowest exponential relaxation and the amplitude growth of the anion twisting mode correlate well, as shown in Figure S3, supporting the assignment of anion anisotropic polarizability relaxation

This interpretation is consistent with the findings of Yamada et al. who demonstrated that in superconcentrated LiTFSI–ACN solutions the TFSI^−^ anion forms extended aggregates with Li^+^ rather than coordinating to ACN [20]. Therefore, the ~25 ps relaxation reflects intrinsic TFSI^−^ polarizability dynamics within the ionic network, not anion–solvent solvation.

Recent research has demonstrated that acetonitrile (ACN) is an efficient solvent for improving ionic conductivity in a range of electrochemical devices, leading to significant interest in the structure of ACN‐based electrolyte solutions [24, 37, 51]. For instance, a 1 mol/L LiTFSI solution in ACN exhibits a conductivity of 36.4 mS/cm, which is significantly higher than values typically reported for many other nonaqueous electrolyte solvents [1]. This improvement in conductivity has been attributed to the reduced viscosity of LiTFSI‐ACN solutions, which can be linked to a lower solvation number [37]. Compared to LiTFSI in other nonaqueous electrolytes solvents, LiTFSI‐ACN electrolytes show less coordination with Li^+^ ions, leading to a higher concentration of free ACN molecules in the electrolyte. Our findings support these conclusions. Additionally, the reduced solvation number in LiTFSI‐ACN is likely due to the weak binding of TFSI anions to Li ions. While ACN has a weaker solvating ability, the loosely bound TFSI anions enable a similar level of salt dissociation in both ACN and other nonaqueous electrolyte systems. Our research, which highlights the dynamic nature of the solvent, adds further evidence that the solvating power of a solvent does not necessarily correlate with the degree of salt dissociation, as other studies have suggested [37]. Furthermore, it is worth noting that our observations bear a conceptual resemblance to the behavior of LHCEs. In LHCEs, the addition of a noncoordinating diluent generates a population of free solvent molecules that decouple bulk viscosity from ion transport. In the case of LiTFSI–ACN, a comparable effect arises naturally: the weak coordinating ability of ACN and the competing coordination of TFSI^−^ anions with Li^+^ ensure that a substantial fraction of ACN molecules remain unbound. These free solvent molecules facilitate enhanced ion conduction despite high salt concentration, thereby mimicking some of the beneficial features of LHCEs without the need for an added diluent [25, 33].

Although the presence of free solvent molecules in concentrated solutions may seem counterintuitive, the primary molecular interactions in these systems are driven by ion–ion interactions, with ion–solvent interactions playing a secondary role. In this scenario, the existence of free solvent molecules supports the concept of intermediate states, where a solvent molecule alternates between coordinating with and dissociating from a lithium ion, effectively hopping between different Li^+^ centers. This interpretation is consistent with the observed rapid coordination and decoordination of solvent molecules in these highly concentrated electrolytes. In addition to the reduced viscosity, the lower Li^+^ coordination number decreases the effective hydrodynamic volume of the solvated species, enhancing translational diffusion coefficients and thereby contributing to the observed high conductivity.

## 3 Conclusion

In this study, LiTFSI‐ACN electrolytes at various concentrations were investigated using OHD‐OKE spectroscopy. This technique, which directly probes the reorientational dynamics of the solvent, offers a more comprehensive and nuanced perspective compared to previous studies. Our results demonstrate that the solvent remains highly dynamic, even at elevated concentrations. These findings support recent explanations for the superior conductivity of acetonitrile‐based electrolyte solutions over other nonaqueous electrolytes. In acetonitrile electrolytes, a higher fraction of free solvent molecules contributes to a significant reduction in viscosity when compared to solvents with equivalent structure. This abundance of uncoordinated solvent molecules facilitates enhanced ion conduction, contrasting with the limited mobility observed in other LiTFSI‐based systems. We stress that this enhanced conductivity cannot be attributed to solvent–anion solvation. ACN does not coordinate TFSI^−^, as confirmed by structural studies. Instead, the slowest relaxation component detected in our OHD‐OKE measurements originates from intrinsic anion polarizability dynamics. As a result, acetonitrile‐based electrolytes offer improved conductivity, making them favorable for applications requiring efficient ion transport. Our findings reveal a dynamical analogy with LHCEs, where free solvent molecules decouple bulk viscosity from ion transport. This conceptual connection is new and provides a framework for understanding why ACN‐based electrolytes maintain superior conductivity at high salt loadings.

While our assignment of relaxation components is based on experimental trends and comparisons with the literature, complementary molecular dynamics simulations would provide microscopic confirmation of the specific solvent–ion and anion dynamics underlying these timescales. Establishing such a combined experimental–computational framework is an important goal for future work. So far, the present study provides the experimental basis for such efforts.

## 4 Experimental Methods

The experimental setups used were similar to those described in previous publications [52]. Briefly, OHD‐OKE is a time‐resolved nonresonant nonlinear optical method in which relaxation of the transient polarizability anisotropy induced by an ultrafast linearly polarized pump pulse is measured. The laser source used here was a commercial titanium sapphire laser (Coherent MIRA 900) with 370 mW output power and a repetition rate of 80 MHz. The pulses were of ~100 fs duration, centered at a wavelength 769 nm. A conventional OHD‐OKE geometry and optical elements were employed [32]. The detected signal, S(t), is a convolution of the solution polarizability response function, R(t), with the instrument response function, $G^{\left(2\right)}(t)$, which is the second‐order autocorrelation of the laser pulses, i.e., $S(t)=R(t)⊗G^{\left(2\right)}(t)$, obtained at the sample position, yielding a FWHM of approximately 140 fs (see figure S4). This width defines the experimental time resolution used in the deconvolution of the OHD‐OKE signal. Full details of the experimental setup and sample preparation are provided in the supplementary information.

## Supporting Information

Experimental method, sample preparation, fitting model, viscosity table, neat ACN OHD‐OKE curve and fit, relaxation and vibration amplitude comparison. Additional supporting information can be found online in the Supporting Information section. **Supporting Fig. S1:** OHD‐OKE signal of neat ACN plus fit (left) and with the underlying exponential components that compose the full fit (right). Bottom, solvent and salt chemical formulas. **Supporting Fig. S2:** Spectral density associated to the OHD‐OKE time domain curves. The weak undulations below ~10 cm^−^¹ arise from the finite experimental time window and apodization of the Fourier transform; they are numerical artifacts rather than physical spectral features. **Supporting Fig. S3:** Blue circles correspond to the amplitude of the fitted 25 ps relaxation component whereas the red square corresponds to the amplitude of the vibrational mode at 120 cm^−1^, which was obtained by adjusting a Gaussian function to this peak for the different curves shown in Figure S2. **Supporting Fig. S4:** The second‐order autocorrelation of the laser pulses was fitted with a Gaussian function, yielding a full width at half maximum (FWHM) of 140 fs. **Supporting Table S1:** Viscosity, density and conductivity for the various solutions that were studied in this work. The values shown in the table were obtained as discussed above, apart from the conductivity, which was retrieved from reference 4. Viscosity and density values measured at 20°C and 25°C (shown); unless otherwise noted, data correspond to 25°C. **Supporting Table** **S2** **:** Summary of global fit parameters (τ _i_,*A*_i_ ) and ±σi standard deviations for each molar fraction. The amplitudes *A*_1_, *A*_2_, *A*_3_ and *A*_4_ correspond, respectively, to the time constants, τ_1_ = 0.48 ps, τ_2_ = 1.65 ps, τ_3_ = 3.70 ps and τ_4_ = 25.0 ps.

## Conflicts of Interest

The authors declare no conflicts of interest.

## Supporting information

Supplementary Material

## Data Availability

The data that support the findings of this study are available from the corresponding author upon reasonable request.
