# Supplementary material for: Ultrafast Kerr Spectroscopy Reveals Bulk‐Like Solvent Dynamics in Concentrated LiTFSI–Acetonitrile Electrolytes
Source: Chempluschem. 2026 Jan 15;91(1):e202500579. doi: 10.1002/cplu.202500579 (PMC12807504; doi:10.1002/cplu.202500579)
Supplement: Supplementary file 1 — Supplementary Material [file CPLU-91-e202500579-s001.pdf]

Ultrafast Kerr Spectroscopy Reveals Bulk-Like Solvent Dynamics in Concentrated LiTFSI–Acetonitrile Electrolytes

Yousaf Shah,[1] Bruno A. Cândido,[2] Pedro Migowski[2], Stephen R. Meech[3] and Ismael A. Heisler,[1]

[1]*Instituto de Física, Universidade Federal do Rio Grande do Sul - UFRGS, Avenida Bento Gonçalves, 9500, Porto Alegre, Brazil*

[2]*Instituto de Química, Universidade Federal do Rio Grande do Sul - UFRGS, Avenida Bento Gonçalves, 9500, Porto Alegre, Brazil*

[3]*School of Chemistry, Norwich Research Park, University of East Anglia, Norwich NR4 7TJ, UK*

Corresponding author: ismael.heisler@ufrgs.br

Experimental Setup

The ultrafast OHD-OKE experiments were performed using a linearly polarized laser pulse with a center wavelength of 769 nm and pulse duration of approximately 100 fs, determined with a home built pulse characterization setup[1]. The ultrafast pulses were generated in commercial Kerr-lens mode-locked Ti:Sapphire laser (Mira 900 Coherent) pumped by 4.60W (CW) intracavity-doubled diode-pumped Nd:YVO_4 laser (Coherent Verdi). The 370 mW femtosecond laser beam was split into probe 5% and pump 95% beams. The pump beam was passed directly through a half wave plate and a polarizer. The probe beam was passed through a computer controlled delay stage (Thorlabs LTS300) to generate a precisely controlled time delay between the probe and pump pulses. A lens with 15 cm focal length was used to focus the two beams on the sample. A phase-sensitive lock-in amplifier, referenced to a chopper that modulates the pump beam at a frequency of 340 Hz, was used to enhance the sensitivity of the detected signal. A balanced detection scheme, using a New Focus 2307 Balanced Photodetector, is employed to get a shot-noise-limited ultrafast birefringence signal as described elsewhere [2]. In this scheme, the probe beam is circularly polarized before the sample with a quarter-wave retardation plate. A Wollaston polarizer is used to separate vertical from horizontal (laboratory reference frame) polarizations of the probe beam. To get the pure heterodyne signal the horizontal is subtracted from the vertical component, to eliminate both homodyne and background components. Additionally, the balanced detection reduces the effects of the laser's power fluctuation and substantially enhances the signal-to-noise ratio.[2]

Sample preparation

Anhydrous acetonitrile (99.8%) and LiTFSI (99.95% trace metals basis) were purchased from Sigma Aldrich and used as received. To prepare the solutions we used the Schlenk technique, first making a highly concentrated solution, with a salt mole fraction of 0.2. The Schlenk flask containing only the salt was heated to 120° C for approximately 24 hours to completely dry the salt. Based on these conditions and the reproducibility of measured viscosities, residual water is expected below 0.01 wt %. Furthermore, the absence of water-related spectral features supports the anhydrous character of the samples. After drying the salt, the Schlenk flask was weighed to determine the exact mass of the salt in the flask. After this anhydrous acetonitrile was added and stirred gently to prepare the solution. For the other mole fractions, this

concentrated solution was diluted with acetonitrile. We made sure that all the flasks are well connected with the Schlenk link for an inert, dry argon atmosphere and anhydrous environment.

Viscosity and Density Measurements

Density and viscosity of nine Schlenk prepared solutions (x_{LiTFSI} = 0.01–0.23) under argon atmosphere, were determined in duplicate using a Stabinger SVM 3000 viscometer (Anton Paar) at 20° and 25° degrees Celsius. Solutions were handled under inert atmosphere and removed through the septum using syringes equipped with stainless steel needles for direct injection into the instrument without contact with the atmosphere. Reported values represent the mean of multiple measurements.

Fitting Model description

The data were fit to a sum of exponentials, given by $(t) = \sum_{i=1}^{n} A_i(x)e^{-t/\tau_i}$. The amplitude $A_i(x)$ is a function of mole fraction. This term is also called the pre-exponential factor. This function was convoluted with the instrument response function, $G^{(2)}(t)$, which is the second order autocorrelation of the laser pulses. The overall fitting function is then given by $S(t) = R(t) \otimes G^{(2)}(t)$. The dynamics acquired through OKE can also be analysed in frequency domain.

The frequency domain representation is readily calculated from the Fourier Transform ($F.T.\{\}$) deconvolution relationship, $.T.\{S(t)\}/F.T.\{G^{(2)}(t)\} = F.T.\{R(t)\} = D(\omega)$. The imaginary part of $D(\omega)$, ($Im\{D(\omega)\}$), contains only information about the nuclear part of the polarisability response. The result is the Raman Spectral Density (RSD= $Im\{D(\omega)\}$),

The kinetics of the data was analyzed by performing a multiexponential fit (global analysis) with the open-source software Glotaran.[3] All fits were carried out over a 0–100 ps time window, beyond which the signal decayed to the noise level.”

Table S1. Viscosity, density and conductivity for the various solutions that were studied in this work. The values shown in the table were obtained as discussed above, apart from the conductivity, which was retrieved from reference 4. Viscosity and density values measured at 20 °C and 25 °C (shown); unless otherwise noted, data correspond to 25 °C

Concentration (mol/L)	x_{LiTFSI}	Viscosity (cP)	Density (g/mL)	Conductivity (mS/cm)
0.00	0	0.35	0.78	0
0.39	0.02	0.36	0.85	26.61
0.90	0.05	0.75	0.95	36.69
1.20	0.07	1.07	1.00	36.38
1.64	0.1	1.57	1.08	32.22
1.93	0.12	2.30	1.13	28.00
2.19	0.14	3.35	1.18	23.51
2.45	0.16	5.16	1.23	18.52
2.91	0.2	12.0	1.31	9.87

Table S2. Summary of global fit parameters (τ_i, A_i) and $\pm\sigma_i$ standard deviations for each molar fraction. The amplitudes A_1, A_2, A_3 and A_4 correspond, respectively, to the time constants, $\tau_1 = 0.48$ ps, $\tau_2 = 1.65$ ps, $\tau_3 = 3.70$ ps and $\tau_4 = 25.0$ ps.

x_{LiTFSI}	A_1	$\pm\sigma_1$	A_2	$\pm\sigma_2$	A_3	$\pm\sigma_3$	A_4	$\pm\sigma_4$
0	0.174	0.0051	0.164	0.001	0	0	0	0
0.02	0.166	0.0071	0.128	0.008	0.016	0.0129	0	0
0.05	0.167	0.0111	0.074	0.009	0.041	0.0141	0.0015	0.0013
0.07	0.165	0.0110	0.052	0.010	0.045	0.0140	0.0033	0.0017
0.10	0.177	0.0118	0.021	0.016	0.052	0.0118	0.0060	0.0030
0.12	0.168	0.0162	0.015	0.013	0.042	0.0122	0.0078	0.0039
0.14	0.162	0.0108	0.011	0.010	0.039	0.0108	0.0093	0.0047
0.16	0.161	0.0107	0.002	0	0.039	0.0097	0.0087	0.0043
0.20	0.174	0.0116	0	0	0.027	0.0071	0.0080	0.0040

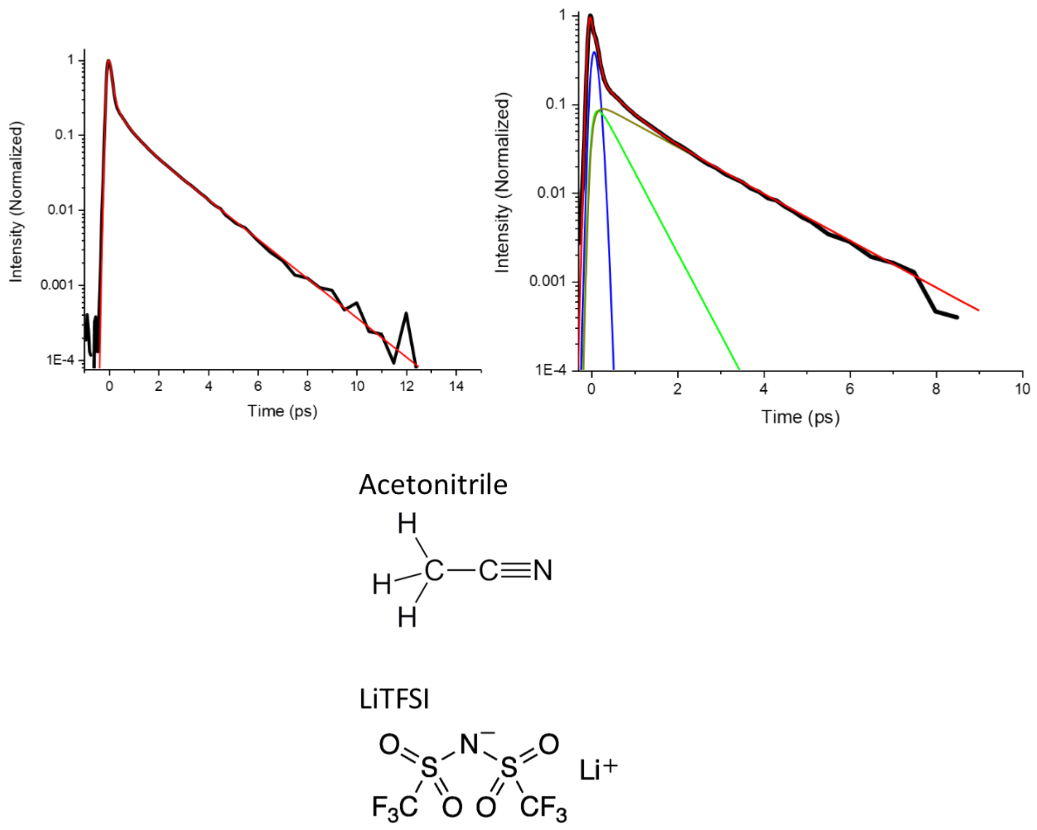

Figure S1. OHD-OKE signal of neat ACN plus fit (left) and with the underlying exponential components that compose the full fit (right). Bottom, solvent and salt chemical formulas.

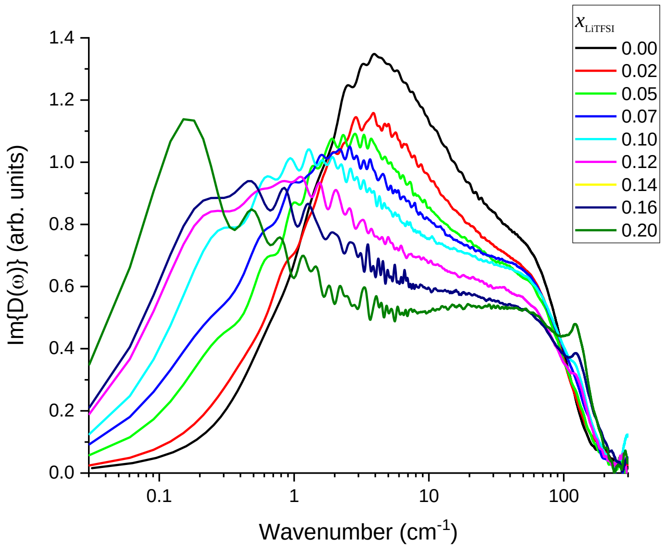

Figure S2. Spectral density associated to the OHD-OKE time domain curves. The weak undulations below ~10 cm^{-1} arise from the finite experimental time window and apodization of the Fourier transform; they are numerical artifacts rather than physical spectral features

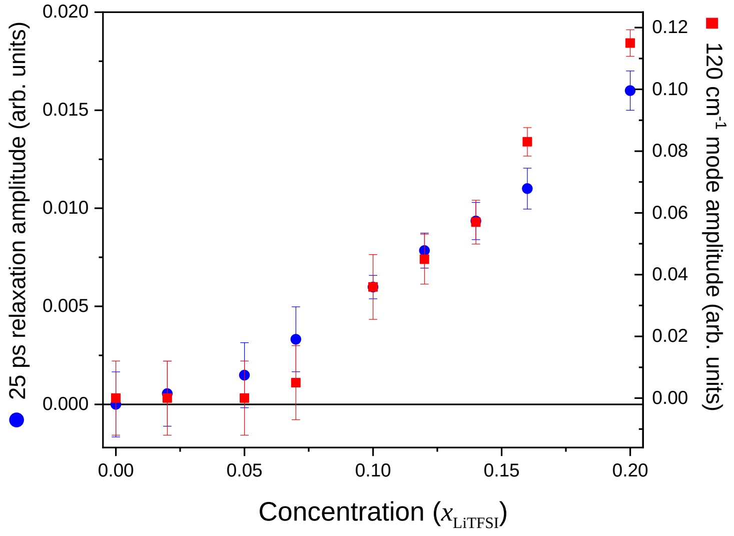

Figure S3. Blue circles correspond to the amplitude of the fitted 25 ps relaxation component whereas the red square corresponds to the amplitude of the vibrational mode at 120 cm^{-1}, which was obtained by adjusting a Gaussian function to this peak for the different curves shown in Figure S2.

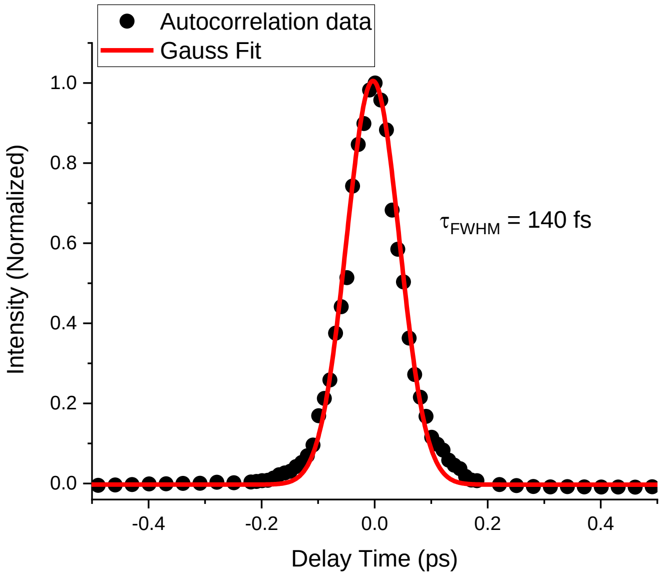

Figure S4. The second-order autocorrelation of the laser pulses was fitted with a Gaussian function, yielding a full width at half maximum (FWHM) of 140 fs.

Reference

(1) Heisler, I. A.; Correia, R. R. B.; Cunha, S. L. S. Characterization of Ultrashort Pulses by a Modified Grating-Eliminated No-Nonsense Observation of Ultrafast Incident Laser Light E Fields (GRENOUILLE) Method. *Appl. Opt.* **2005**, *44* (16), 3377–3382..

(2) Turton, D. A.; Martin, D. F.; Wynne, K. Optical Kerr-Effect Study of Trans- and Cis-1,2-Dichloroethene: Liquid-Liquid Transition or Super-Arrhenius Relaxation. *Phys. Chem. Chem. Phys.* **2010**, *12* (16), 4191–4200.

(3) Snellenburg, J. J.; Laptenok, S. P.; Seger, R.; Mullen, K. M.; Van Stokkum, I. H. M. Glotaran: A Java-Based Graphical User Interface for the R Package TIMP; *Journal of Statistical Software* **2012**; *49* (3) 1-22.

(4) Koo, B.; Hwang, S.; Ahn, K. H.; Lee, C.; Lee, H. Low Solvating Power of Acetonitrile Facilitates Ion Conduction: A Solvation-Conductivity Riddle. *J. Phys. Chem. Lett.* **2024**, 3317–3322. https://doi.org/10.1021/acs.jpclett.4c00545.
